# *Wolbachia* infection and Lepidoptera of conservation concern

**DOI:** 10.1093/jis/14.1.6

**Published:** 2014-01-01

**Authors:** C. A. Hamm, C. A. Handley, A. Pike, M. L. Forister, J. A. Fordyce, C. C. Nice

**Affiliations:** 1 Department of Entomology and Program in Ecology, Evolutionary Biology and Behavior, Michigan State University, East Lansing, Michigan 48823; 2 Department of Biology, Population and Conservation Biology Program, Texas State University, San Marcos, Texas 78666; 3 Department of Biology, University of Nevada Reno, Reno, Nevada 89557; 4 Department of Ecology and Evolutionary Biology, University of Tennessee, Knoxville, Tennessee 37996; 5 Department of Evolution and Ecology, University of California-Davis, One Shields Avenue, Davis, California 95616; 6 Department of Molecular Microbiology and Immunology, Johns Hopkins University, Baltimore, Maryland 21205

**Keywords:** captive rearing, endangered species, endosymbiont, translocation

## Abstract

Conservation of at-risk species requires multi-faceted and carefully-considered management approaches to be successful. For arthropods, the presence of endosymbiotic bacteria, such as *Wolbachia* (Rickettsiales: Rickettsiaceae), may complicate management plans and exacerbate the challenges faced by conservation managers. *Wolbachia* poses a substantial and underappreciated threat to the conservation of arthropods because infection may induce a number of phenotypic effects, most of which are considered deleterious to the host population. In this study, the prevalence of *Wolbachia* infection in lepidopteran species of conservation concern was examined. Using standard molecular techniques, 22 species of Lepidoptera were screened, of which 19 were infected with *Wolbachia* . This rate is comparable to that observed in insects as a whole. However, this is likely an underestimate because geographic sampling was not extensive and may not have included infected segments of the species’ ranges. *Wolbachia* infections may be particularly problematic for conservation management plans that incorporate captive propagation or translocation. Inadvertent introduction of *Wolbachia* into uninfected populations or introduction of a new strain may put these populations at greater risk for extinction. Further sampling to investigate the geographic extent of *Wolbachia* infections within species of conservation concern and experiments designed to determine the nature of the infection phenotype(s) are necessary to manage the potential threat of infection.

## Introduction

Conservation managers often adopt active management strategies when confronted by complex and interconnected problems. Captive propagation and translocation programs are increasingly being incorporated into management plans to augment endangered populations or repopulate formerly occupied habitats ( Crone et al. 2007 ). These programs present their own challenges and must be carefully designed to minimize the possibility of disease transmission and maintain genetic diversity ( Snyder et al. 1996 ; Van Oosterhout et al. 2007 ). When working with arthropods of conservation concern, an additional and under-appreciated challenge arises in the form of endosymbiotic bacteria that may manipulate the reproductive biology of their hosts (Werren et al. 2008; Nice et al. 2009 ).

The α-proteobacteria *Wolbachia* (Rickettsiales: Rickettsiaceae) is a maternally inherited endosymbiont of many arthropods and is estimated to occur in up to 66% of all insects ( Hilgenboecker et al. 2008 ). *Wolbachia* has been the focus of intense research efforts due to its potentially significant effects on the reproduction of its host ( Werren et al. 2008 ; Nice et al. 2009 ). In short, *Wolbachia* manipulates its host’s reproduction to facilitate its own by inducing one of four phenotypes: (1) feminization occurs when a *Wolbachia* infection transforms genetically male embryos into fully functional females, leading to production of progeny that are all functionally female; (2) male-killing strains eliminate all male embryos so only female progeny are produced; (3) parthenogenesis-induction occurs in species with haplodiploid sex determination in which infected females do not need to mate and therefore produce only female progeny from unfertilized eggs; (4) cytoplasmic incompatibility (CI) prevents infected males from reproducing with uninfected females or females infected with a different strain of *Wolbachia* , and this is perhaps the most common phenotype ( Werren et al. 2008 ). Should CI-infected individuals be introduced to an uninfected populations, the consequences may be dire, especially for small populations ( Nice et al. 2009 ). These CI infections may induce a population bottleneck as its frequency spreads; if the population is not large enough to pass through this bottleneck, it may be extirpated. *Wolbachia* infection can be difficult to detect by demographic observations, and laboratory experiments to determine infection phenotype can be costly and time consuming ( Weeks et al. 2007 ). The most common method for detecting *Wolbachia* infection utilizes the cost effective PCR, and strains may be identified using standardized protocols ( Baldo et al. 2006 ).

Within the insects, Lepidoptera are disproportionately represented among the threatened and endangered species in the United States (28 of 62 species; U.S. Fish &Wildlife Service). Lepidoptera have also been the subject of captive rearing programs and have been discussed as candidates for translocation (Tolson 2008; Richardson et al. 2009 ; Landis et al. 2011 ). While the threat of *Wolbachia* to Lepidoptera of conservation concern has been demonstrated theoretically ( Nice et al. 2009 ), it remains to be seen if *Wolbachia* infections are present in Lepidoptera of conservation concern. Therefore, 22 lepidopteran species from the United States, including federal and state listed threatened and endangered taxa, were surveyed for *Wolbachia* to document the presence of the bacterium and draw attention to the role that infection may play in recovery efforts.

## Materials and Methods

A total of 296 individuals from 22 at-risk lepidopteran species were sampled ( Table 1 ). The very nature of threatened and endangered species prohibits the use of large and random samples, thus these taxa were utilized because the material was either at immediate disposal or was kindly donated by colleagues. Genomic DNA (gDNA) was extracted from all individuals following standard methods ( Brookes et al. 1997 ). Screening for *Wolbachia* in gDNA samples required two PCRs and followed the methods used by Nice et al. (2009) . Primers for the ribosomal rDNA gene 16S (WSpecF and WSpecR) were used to detect *Wolbachia* and were run in concert with arthropod-specific 28S rDNA primers (28sF3633 and 28sR4076) to act as a positive control for each reaction. Standard positive and negative controls were run simultaneously during the *Wolbachia* screens. PCR products were visualized on a 1% agarose gel and scored for the presence of *Wolbachia* .

**Table 1.**
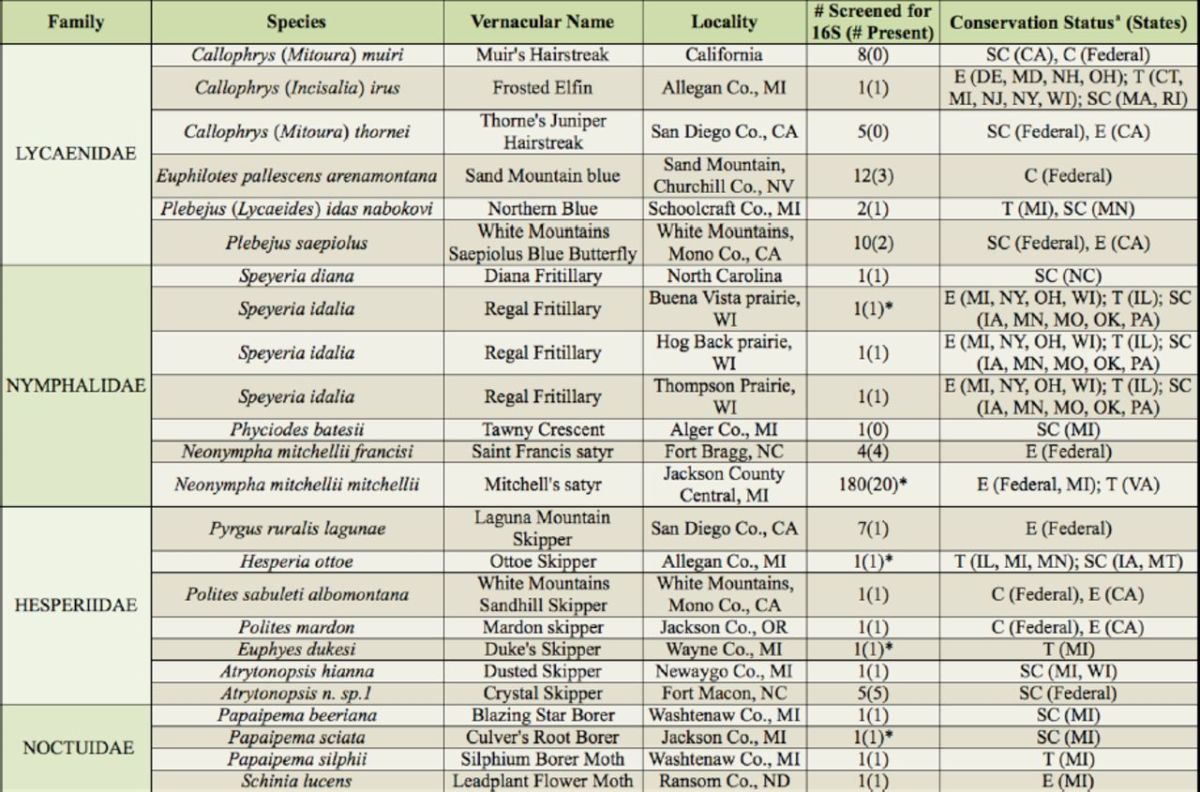
Results of a survey of *Wolbachia* infections in 244 lepidopteran individuals, representing 22 species of conservation concern. The county of collection was not available for some individuals. The total number screened is presented with the number testing positive for *Wolbachia* presence, which is given parentheses. All samples that were positive for 16S also tested positive for *fbpA* .

^a^ Correspoding codes: T = threatened; E = endangered; SC = species of special concern; C = candidate for protection; * = sequenced

While the use of *Wolbachia* -specific PCR remains the standard method for the detection of *Wolbachia* ( Baldo et al. 2006 ), certain primers may be prone to cross amplification of other bacteria ( Simões et al. 2011 ). To minimize the likelihood of false positives, additional primers were used to amplify fructose-bisphosphate aldolase ( *fbpA* ) for a subset of 16 individuals that tested positive for 16S ( Baldo et al. 2006 ) ( Table 1 ); *fbpA* amplification followed the PCR protocols outlined in Simões et al. (2011) . This combination of molecular markers, 16S, and *fbpA* should only amplify *Wolbachia* and no other bacterial endosymbiont ( Simões et al. 2011 ). A subset of individuals that tested positive for *Wolbachia* infections were sequenced in both directions for 16S and *fbpA* at the Research Technology Support Facility at Michigan State University using an ABI Prism 3730 Genetic Analyzer (Applied Biosystems, Life Technologies, www.lifetechnologies.com ). The resulting sequences were combined into contigs using Geneious version 5.4 (Biomatters, www.biomatters.com ) and submitted for BLAST search to confirm the amplification of *Wolbachia* sequences ( Altschul et al 1990 ).

To further minimize the chances of false positives for individuals that were not sequenced, a restriction digest was conducted. It was possible to predict the size of DNA fragments generated by certain restriction enzymes using the genetic sequences previously produced. The 4-cutter *MseI* (New England Biolabs, www.neb.com ) should have two restriction sites in 16S, both at the ends of the sequence, and generate one band of approximately 300 bp and two bands of 100 bp. The gene *fbpA* should contain two restriction sites for *MseI* , both located at the ends of the sequence, and would generate one band of approximately 350 bp and two bands of 75bp. Restriction digests were carried out under manufacturer’s specifications and the resulting products were visualized on a 2% agarose gel to confirm that the actual band sizes matched predictions.

## Results

19 of the 22 species screened had at least one individual score positive for *Wolbachia* infection, and a subset of individuals positive for 16S were also positive for *fbpA* . Individuals from six species ( *Neonympha mitchellii mitchellii* French (Nymphalidae), *Speyeria idalia* (Drury), *S. diana* (Cramer), *Eupheys dukesii* (Lindsey) (Hesperiidae), *Hesperia ot-toe* Edwards, and *Papaipema sciata* Bird (Noctuidae)) were sequenced for 16S and *fbpA* , resulting in reads of ~400 bp in length. All sequences queried with a BLAST search returned hits to the appropriate *Wolbachia* isolate with 100% pairwise identity (E value = 0) and all sequences were associated with *Wolbachia* strains from supergroup 2 (Gen-Bank accession numbers KJ125429– KJ125436). Samples subjected to *MseI* restriction digest produced bands that matched the length predicted by restriction site mapping to known sequences for 16S and *fbpA* .

## Discussion

The presence of *Wolbachia* was documented in 19 lepidopteran species of conservation concern, one of which ( *N. m. mitchellii* ) is the focus of captive rearing efforts ( Tolson 2008 ). Multiple markers were utilized, a subset of individuals was sequenced, and restriction digests were conducted on the remaining individuals to minimize the possibility of false positives. However, caution is urged when interpreting these results because a small sample size was used for many taxa and the data were not randomly sampled (though the conservation status of these taxa precludes random sampling). Thus, while these species tested positive for infection, the limited sampling reported here cannot be extended to the entire range of species. Furthermore, geographically comprehensive sampling would be required for any management plans for infected species.

Accurately determining the presence of infection and its induced phenotype requires the use of multiple methods. While necessary to detect the presence of infection, molecular genetic tools alone cannot identify the induced phenotype, because induced phenotypes are not monophyletic and the same strain might induce different phenotypes in different species ( Zhou et al. 1998 ; Baldo et al. 2006 ). Molecular methods must be used in conjunction with demographic observations to determine the effects of *Wolbachia* . Sequencing alone cannot allay concerns regarding the effects of *Wolbachia* . For example, *Wolbachia* from the *Culex pipiens / quinquefasciatus* complex are identical at multiple loci, yet crosses between some strains result in cytoplasmic incompatibility ( Duron et al. 2006 ; Atyame et al. 2011 ). Indeed, even utilizing the MLST protocol, in which five variable *Wolbachia* loci are sequenced, may or may not distinguish strains ( Baldo et al. 2006 ). The *Wolbachia* from the invasive vinegar fly *Drosophila suzukii* is identical to *wRi* (the strain found in *Drosophila simulans* ) at all five MLST loci, and yet had polymorphisms at the *dnaA* locus and numerous inversions; these differences were only observed after genomic sequencing was conducted ( Siozios et al. 2013 ). Confirming infection by a CI strain requires experimental crosses between infected and uninfected individuals. In addition to *Wolbachia* , there are a number of other reproductive manipulators associated with insects, such as *Cardinium* , *Rickettsia* , and *Spiroplas-ma* , that should also be screened for and considered ( Duron et al. 2008 ).

Assuming deleterious effects of an infection, there are two additional concerns when managing *Wolbachia* : (1) the possibility that the induced phenotype is suppressed ( Hornett et al. 2006 ), and (2) the possibility that there are multiple strains occurring sympatrically in the same population ( Hiroki et al. 2004 ). In the case of suppressed phenotypic effects, some populations may appear uninfected because they have been able to ameliorate the phenotypic effects of infection, though they remain infected. These populations have been able to suppress the infection phenotype, and while *Wolbachia* are still detectable using molecular methods, the infection phenotype is absent ( Hornett et al. 2006 ). Transmission of *Wolbachia* from a suppressing population to an uninfected population may result in the expression of the induced phenotype and all of the subsequent consequences. While suppression might allow populations to escape the consequences of infection, the concealed presence of *Wolbachia* increases the likelihood of inadvertently introducing an infection through captive management or translocation. In the case of infection by multiple strains of *Wolbachia* ( Reuter and Keller 2003 ; Hiroki et al. 2004 ), each strain might induce a different phenotype.

For these reasons, management programs should screen a representative subset of individuals propagated in captivity to verify if infection is present. This study may serve as a foundation for examining other at-risk arthropods, many of which have captive propagation programs. Screening of these species is necessary to determine the extent of *Wolbachia* infection within populations and across species. Future studies should also seek to determine the nature of the *Wolbachia* -induced phenotypes in these species. Information on the prevalence, geographic extent, and phenotypic effects of *Wolbachia* might prove critical for effective management of threatened and endangered arthropods.

## Abbreviations

CI: cytoplasmic incompatibility
fbpA: fructose-bisphosphate aldolase
